# Malakoplakia mimicking malignant rectal carcinoma with bone erosion in a SLE patient: diagnosis insights from a case report and literature review

**DOI:** 10.3389/fimmu.2025.1542777

**Published:** 2025-05-21

**Authors:** Lingshu Zhang, Jingyao Zhang, Ji Wen, Hang Yang, Ling Sun, Tianwen Huang, Yixin Sun, Wei Jiang, Chunyu Tan

**Affiliations:** ^1^ Department of Rheumatology and Immunology, West China Hospital, Sichuan University, Chengdu, Sichuan, China; ^2^ Department of Core Facilities, West China Hospital, Sichuan University, Chengdu, Sichuan, China; ^3^ Department of Radiology, West China Hospital, Sichuan University, Chengdu, Sichuan, China; ^4^ West China School of Medicine, Sichuan University, Chengdu, Sichuan, China; ^5^ Department of Traditional Chinese Medicine, University-town Hospital of Chongqing Medical University, Chongqing, China

**Keywords:** malakoplakia, SLE, Michelis Gutmann body, bone erosion, colon

## Abstract

Malakoplakia, an uncommon granulomatous inflammatory disease, is frequently associated with impaired immune function in adults, and its occurrence in patients with systemic autoimmune diseases such as SLE has not been widely studied. The variable clinical manifestations and sometimes non - specific radiological findings of malakoplakia can be misleading, thereby posing a challenge for diagnosis. We report a case of colon and bone malakoplakia mimicking malignant colonic carcinoma in an 18-year-old male with systemic lupus erythematosus (SLE). This case illustrates the diagnostic challenges associated with malakoplakia in patients with SLE. The presence of non-specific symptoms and the mimicry of more common and serious conditions can lead to delayed diagnosis and inappropriate management. Clinicians should be aware of the potential for unusual infectious processes in patients with rheumatic diseases, especially when dealing with abscess-like symptoms. Understanding the pathophysiology and clinical features of malakoplakia in these patients is essential for accurate diagnosis and timely intervention.

## Introduction

Malakoplakia is a rare granulomatous inflammatory disease thought to be secondary to a defect in macrophage bactericidal activity. The etiology of this condition has not been fully elucidated, but it is typically associated with immunocompromise. Malakoplakia most commonly affects the urinary tract, but involvement of various organ systems such as the digestive tract (esophagus, colon, rectum), female reproductive system (breast, endometrium, cervix), lungs, kidneys, skin, and brain has also been reported. Malakoplakia frequently involves the mucosal surface, presenting as single or multiple papules, plaques, ulcers, or even local neoplasms. Radiologists and clinicians may mistake it for a malignant lesion, leading to incorrect diagnosis and treatment. We present a case of malakoplakia in a patient with systemic lupus erythematosus (SLE), causing both digestive symptoms and bone erosion that mimicked malignant rectal carcinoma. Clinicians should be aware of this rare manifestation in immunocompromised patients.

## Case presentation

An 18-year-old male presented with constipation for 2 weeks and was referred to our hospital for further evaluation on January 20, 2023. He had been diagnosed with SLE in 2020 and was on a maintenance dose of prednisone 15mg once daily and hydroxychloroquine 200mg once daily for several years. He had a history of herpes zoster six months ago. On admission, his vital signs were as follows: body temperature 36.3°C; blood pressure 128/89 mmHg; respiratory rate 22 breaths per minute; and pulse rate 120 beats per minute. Physical examination was unremarkable, except for a small amount of pigmentation on his left lower back. There were no signs of alopecia, rashes, photosensitivity, or oral ulcers. Laboratory studies revealed the following: hemoglobin 103 g/L (130-175); white blood cell (WBC) count 18.71×10^9^/L (3.5-9.5); platelet count 409×10^9^/L (100-300), with 93.4% neutrophils (40-75); C-reactive protein 98.70 mg/L (0-5); erythrocyte sedimentation rate (ESR) 74.0 mm/h (<21); interleukin-6 (IL-6) 126.10 pg/ml (0-7). Renal function was normal. Urinalysis showed red blood cells (RBC) 168/uL, white blood cells (WBC) 31/uL, urine protein 0.3 (1+) g/L, epithelial cells 13/uL, WBC 6/HP, and RBC 30/HP (0-11). Urinary RBC morphology examination revealed RBCs +/HP, and 100% abnormal red blood cells, with wrinkled and small red blood cells visible. The 24-hour urine protein content was 0.29 g/24h. Stool culture showed WBCs 3-5/HP, RBCs 0-1/HP, pus cells detected/HP, and occult blood (immunoassay) was positive. The antinuclear antibody titer was 1:1000, anti-dsDNA 46.90 IU/ml (<30), autoantibodies to ribosomal P proteins positive, anti-proteinase 3 antibody 49.70 AU/ml (<20), with elevated IgM 4480 mg/L(700-2200), and complement factor consumption complement C3 0.5650 g/L (0.785-1.52), complement C4 0.0507 g/L (0.145-0.36), rheumatoid factor (RF) 20.00 IU/ml (0-20); CD3 absolute count 590 cells/ul (941-2226), CD4 absolute count 282 cells/ul (471-1220), CD8 absolute count 301 cells/ul (303-1003), B cell absolute count 19 cells/ul (175-332), NK cell absolute count 83 cells/ul (154-768). Anti-CCP antibodies, anti-keratin antibodies, anti-cardiolipin antibodies, and lupus anticoagulant were negative. Epstein-Barr virus <50 copies/mL, and interferon-gamma release assays showed no abnormalities.

**Figure 1:**
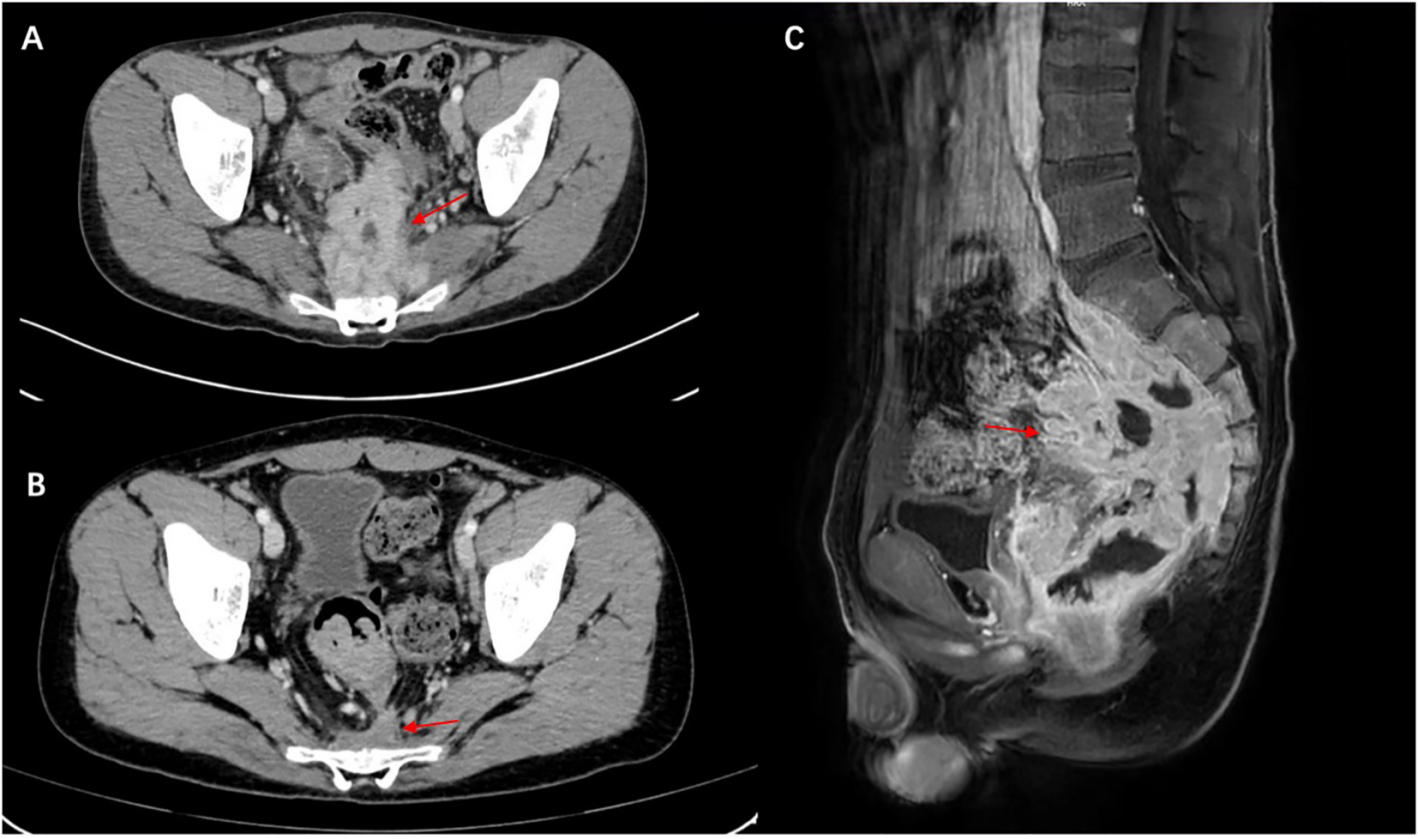
Malakoplakia in the presacral space on computed tomography scan and MRI. **(A)** Before treatment and **(B)** the presacral space soft tissue mass has markedly decreased after treatment (red arrow). **(C)** MRI scan shows a large mass of soft tissue in front of the sacrum (red arrow), with destruction of the vertebral body bone before treatment.

Abdominal ultrasonography revealed a low echogenic mass in the pelvic cavity, approximately 8.8 x 6.4 x 8 cm in size. The boundary was unclear, the shape was irregular, and the internal echo was uneven. Dotted linear blood flow signals could be seen inside. Abdominal computed tomography (CT) showed a soft tissue density mass in the anterior sacral space (approx. 8.5 × 6 × 9.2 cm), with multiple enlarged lymph nodes, thickening of the fascia around the sacrum and rectum, pelvic floor fascia, and slight bone destruction of the adjacent sacral vertebrae, and a small amount of fluid accumulation in the pelvic cavity (**Figure 1A**). Thoracolumbar spine Magnetic Resonance Imaging (MRI) revealed bone destruction of the sacral 1, 2, and 3 vertebral bodies, with large soft tissues in the anterior sacrum resembling pelvic cavity involvement, suggesting the possibility of granulomatous infectious lesions, but lymphoma needs to be excluded. MRI pelvic enhancement scan was performed showing irregular mass shadows can be seen in the surrounding space of the rectum, involving the sacral vertebrae, sacral canal, and left second sacral foramen, considering the possibility of granulomatous infectious lesions or other factors, as well as multiple enlarged lymph nodes in the mesorectum and pelvic cavity. Blurred left sacroiliac joint surface suggested secondary inflammatory changes? Abnormal signal of right iliac wing, front edge of neck of femur and acetabulum, infection? Simple bone marrow edema? Or other needed to be excluded (**Figure 1C**).

The patient underwent Flu-deoxy-glucose Positron Emission Tomography (FDG-PET) scan for overall assessment. PET-CT revealed rectal wall thickening, local intestinal dilation, and a soft tissue mass with heterogeneous density anterior to the sacrum. The local boundary with the sigmoid colon, prostate, left seminal vesicle gland, and sacrum was indistinct, and the surrounding fat space was blurred, with a larger section of approximately 71 × 62 mm and abnormal 18F-FDG uptake. Local invasion of the adjacent sacrum, involvement of the left side of the sacral canal at the level of sacrum 2, bilateral sacral foramina at sacrum 1, and left sacral foramen at sacrum 2 were noted, with a maximum SUV of 14.76.

**Figure 2:**
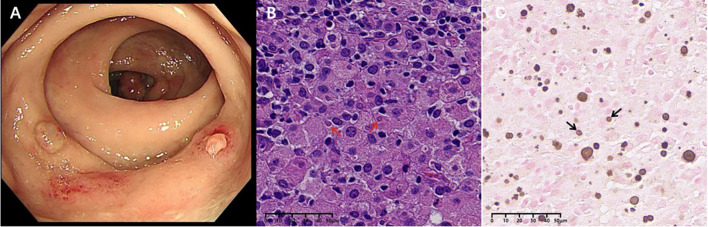
Rectal mucosa: **(A)** Colonoscopy suggests colonic polyps and rectal nodular elevations. **(B)** MG bodies (red arrows) were seen in Von Hansemann cells (HE staining, X400). **(C)** Calcium salt staining with label X (von Kossa silver nitrate method) showed calcium salt deposits in MG bodies (black arrows).

**Figure 3:**
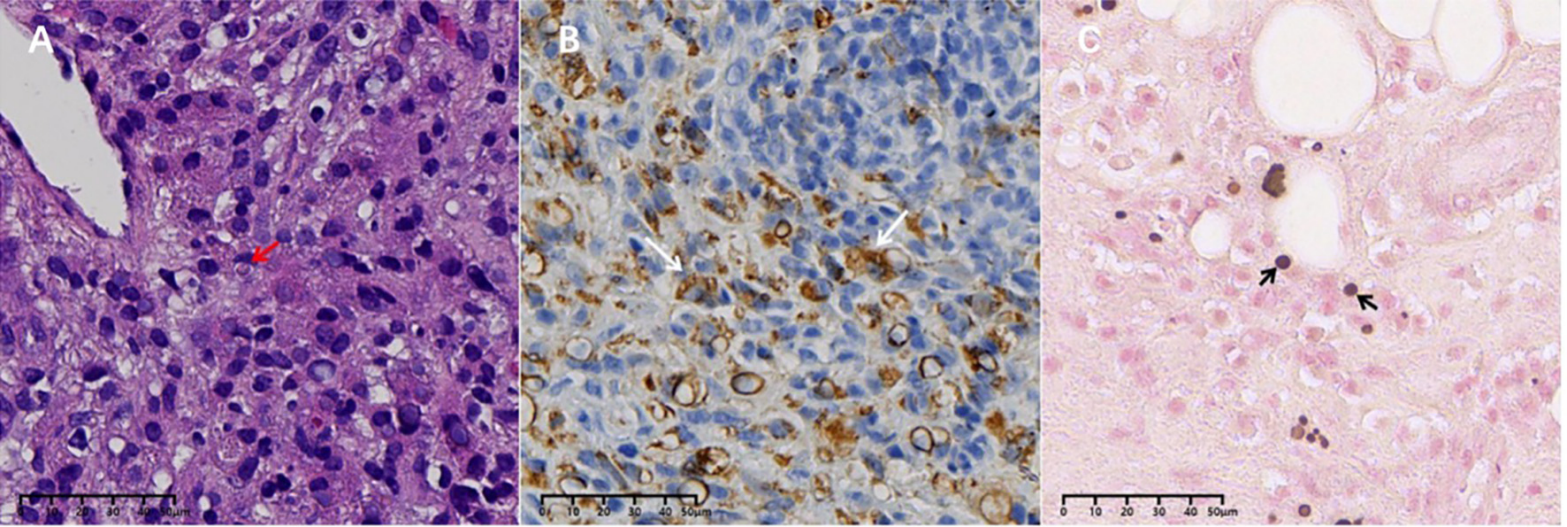
Presacral mass: **(A)** MG bodies (red arrows) were seen in some tissue cells (HE staining, X400). **(B)** CD68-positive cells of the monocyte/macrophage lineage stained with CD68 (white arrows) (×400). **(C)** Calcium salt staining (von Kossa silver nitrate method) showed calcium salt deposits in MG bodies (black arrows).

We informed the patient of the importance of tissue biopsy for a more accurate diagnosis. After obtaining written consent for the surgery, the biopsy of the ascending colon revealed mucosal histiocyte hyperplasia and aggregation, and Michelis Gutmann (MG) bodies were found in the cytoplasm of individual cells (**Figure 2**). To further clarify the nature of the presacral mass and rule out malignant lesions, histology of the presacral mass suggested fibrous tissue hyperplasia and chronic inflammatory cell infiltration, with active focal cell proliferation. Immunohistochemistry demonstrated positivity for CD68 (PGM-1), SMA, and CD163, with preserved INI-1 expression and a Ki-67 proliferation index of 5%. All other lineage markers (SOX2, ALK-1, CD31, CD34, Desmin, PCK, S-100, CD1a, Langerin, myogenin, myo D1) were negative. Histochemical studies revealed calcium deposits (calcium stain +) and PAS-positive material, while stains for iron (Prussian blue), mycobacteria (AFB), fungi (GMS), and intracellular pathogens (Giemsa) were negative. Characteristic Michaelis-Gutmann bodies within histiocytes confirmed the diagnosis of malacoplakia (**Figure 3**). Following a comprehensive examination with the pathological findings, the final diagnosis of malacoplakia with bone erosion was made.

The patient was initiated on oral levofloxacin with prednisolone 10mg/day and hydroxychloroquine 200mg once daily. We advised the patient to gradually taper the drugs over the next 6 months. Since then, the patient has shown good improvement. At the 72-week follow-up, the patient did not complain of constipation. Repeat CT scans of the chest revealed good resolution of the lesion as well (**Figure 1B**). He was also advised to undergo regular follow-up for abdominal examinations to detect early recurrence or progression of the disease.

## Discussion

Professor von Hanseman first coined the term “malakoplakia” in 1901, while Leonor Michaelis and Carl Gutmann published the first paper on the condition in 1902 (1, 2). The term comes from the Greek “malakos” (soft) and “plakos” (plaque), describing the clinical appearance of the lesions as friable white-yellow soft plaques raised on the mucosal surface. The principal microscopic feature is infiltration of large granular macrophages termed Hansemann histiocytes or von Hansemann cells, followed by intracellular deposition of iron and calcium known as Michaelis-Gutmann (MG) bodies.

The pathogenesis remains incompletely understood. The primary putative pathologic mechanism involves impairments in the phagocytic or degradative functions of histiocytes in response to gram-negative coliforms. *Escherichia coli* (*E. coli*) is the most prevalent causative species associated with this condition; however, other organisms such as *Klebsiella*, *Proteus*, *Corynebacterium*, *Pseudomonas*, *Acinetobacter*, *Staphylococcus*, *Streptococcus*, *Enterococcus*, *Aerobacter*, *Rhodococcus*, *Mycobacterium*, *Salmonella*, and *Burkholderia* species have also been implicated (3–5).

Several defects in mononuclear phagocyte function may predispose to the development of malakoplakia. Firstly, macrophages are sentinel cells representing the first line of defense against invading bacterial pathogens. Once encountered, extracellular bacteria are internalized into phagosomes, which are delivered to endolysosomes, and effective clearance of bacteria relies on subsequent lysosomal degradation. Improperly functioning lysosomes that do not release sufficient β-glucuronidase may result in malakoplakia. β-Glucuronidase and cyclic guanosine monophosphate (cGMP) are required for normal microtubular function and phagolysosomal activity, and dysfunction may lead to the accumulation of partially digested bacteria in macrophages, causing malakoplakia (6).

Human β-glucuronidase is homologous to the β-galactosidase enzyme found in *E. coli* (7). Secondly, decreased levels of cGMP have been demonstrated in patients with malakoplakia compared to healthy controls (8). This is believed to lead to decreased clearance of pathogenic organisms due to the persistence of phagolysosomes. Thirdly, certain organisms of *Stenotrophomonas bacteremia* are inclined towards biofilm formation, potentially resulting in iron deposition and aggregation of the organism within the hepatic macrophages (9). However, Hyun et al. reported a healthy 19-year-old female who developed colonic malakoplakia without finding exact microorganisms in the stool and blood culture. Therefore, other factors must contribute to this pathological process (10).

Malakoplakia has been documented in patients ranging in age from fetus to 88 years in various recesses (7, 11), predominantly in the adult population with a mean age at diagnosis in the fifth decade, while cases in pediatric patients are relatively rare. Literature review emphasizes that malakoplakia favors to develop as a comorbidity in immunocompromised hosts with primary or secondary immunodeficiency states. There are very few reported cases documented in the pediatric population. Beginning at an early age, these patients presented with various primary immune defects such as X-linked agammaglobulinemia, common variable immune deficiency, severe combined immunodeficiency (7), and primary hypogammaglobulinemia (12). Malakoplakia is a rare condition with only a few reported cases in acquired immunodeficiency syndrome (HIV) (13, 14). Due to immunodeficiency causing impeded macrophage and T cell function, most patients experience intermittent or even persistent infections, which predispose to the development of malakoplakia. The coexistence of significant rheumatic diseases or their treatment and malakoplakia has been noted in a few cases, such as SLE (8, 15, 16), rheumatoid arthritis (RA), Sjögren’s syndrome (17), polymyositis (18) and systemic sclerosis (19).

Only 8 case reports were associated with SLE, including our case. (**Table 1**) There is a 1:1 female to male predilection, with an age range from 16 to 58 years old, and a mean age at diagnosis of 38 years old. Malakoplakia shows a variable course, with a mean duration of disease ranging from 18 weeks after kidney transplantation to 22 years. The genitourinary system, such as the kidney, is the most frequent localization, followed by the gastrointestinal tract. However, in our case, there were two different sites involving both the colon and the sacrum. *E. coli* is the most common causative species of bacteria isolated from these patients, while other organisms include *Stenotrophomonas maltophilia*, *Enterococcus faecium*, *Acinetobacter calcoaceticus*, and *Pseudomonas aeruginosa*. Interestingly, the female urinary and reproductive systems are more susceptible to infection due to physiological factors, and continuous irritation of the urinary system may potentially open the window to initiate the inflammatory process and progressively develop malakoplakia. Azathioprine (AZA) plus prednisone was the immunosuppressive regimen used in 3/8 patients (18, 20, 21). Indeed, for SLE itself, it has been established that macrophages affect the development of lupus (22). A defect in macrophages leads to inefficient digestion of phagocytosed contents and results in increased secretion of proinflammatory cytokines and decreased secretion of anti-inflammatory cytokines (23). Furthermore, as an orally administered drug, AZA has a complex metabolic pathway with the microbiota. One study has examined the interaction between the effects of AZA, 6-mercaptopurine (MP), and 5-aminosalicylic acid on the growth of IBD-associated bacterial species and identified bacterial enzymes involved in immunosuppressive drug metabolism. The growth of *E. coli* was only significantly inhibited by 200 μg/ml of AZA (24). Nevertheless, the therapeutic response and variability of genetic polymorphism of the enzyme are highly variable among individuals (25). Glucocorticoids (GCs) are highly potent anti-inflammatory drugs that suppress macrophage activity, especially in the first-line therapy for life-threatening conditions such as macrophage activation syndrome. Mycophenolate mofetil is a relatively novel immunosuppressant that inhibits macrophage infiltration and kidney fibrosis (26). However, Boo et al. reported a patient with lupus-related end-stage renal disease and experienced post-transplantation malakoplakia. Interestingly, it developed in the native kidney but not in the graft one (16). The long-term effects of steroids and immune-suppressive medication on macrophage interaction remain unclear.

**Table 1:** Case reports of malakoplakia in SLE.

Age/sex	Immune status	Duration before Malakoplakia Diagnosis	Location	Infection	Treatment	Outcome	Reference
42/M	SLE	2 years	Axilla	*E. coli*	Steroids and AZA	Recovered	(18)
53/M	SLE and chronic refractory pancytopenia	6 month	Liver	*Stenotrophomonas maltophilia, Enterococcus faecium*, and *invasive pulmonary aspergillosis.*	Steroids	Dead	(9)
54/F	SLE	2 years	Liver	*E.tcherichia coli* and *Acinetobacter calcoaceticus.*	Steroids and AZA	Dead	(20)
16/F	SLE	4 years	Kidney	*E. coli*	Steroids	Recovered	(8)
25/F	SLE LN and adrenogenital syndrome	2 years	Kidney	*E. coli, Pseudomonas aerugino*	Steroids and AZA	Dead	(21)
40/F	SLE LN and kidney transplant	18 weeks after transplantation.	Kidney	*E. coli*	Tacrolimus, steroids, and MMF	Recovered	(16)
58/M	SLE LN and Sjögren's syndrome	22 years	Colon	Not mentioned	MMF, rituximab, Tacrolimus, steroids and hydroxychloroquine	Recovered	(15)
18/M	SLE	3 years	Colon and sacrum	*E. coli*	Steroids and hydroxychloroquine	Recovered	

*E. coli*, *Escherichia coli*; AZA, azathioprine; MMF, mycophenolate mofetil; lupus nephritis, LN.

Malakoplakia can often clinically mimic malignancy and result in an abscess-like effect (27, 28). However, malakoplakia has been described in the literature as a benign disease process typically presenting in middle-aged patients. The radiographic appearance is usually tumor-like, and given its rarity, it is solely based on histological analysis. The need to await a surgical biopsy and pathological diagnosis is crucial to ensuring a correct diagnosis. Bone is an uncommon site for malakoplakia. Lane et al. reviewed previous cases of malakoplakia of the bone identified in the literature (29). The most common presenting symptoms were pain, swelling, and some even had a pathologic fracture at the site of involvement. Four of the nine cases demonstrated right femur involvement, and the distal region such as the skull had also been reported. *E. coli* was isolated in five of the nine cases involving bone. Additionally, some of these patients have an immunosuppressed background, possibly of *E. coli* origin from the colon, which may play an etiological role in causing osteomyelitis.

It appears that our patient and those described previously are different. This young patient entered remission after long-term treatment with low-dose prednisone and hydroxychloroquine. Thus, malakoplakia may be associated with the clinical immune status, but not in all cases. Furthermore, he complained of constipation for 2 weeks, and typically, *E. coli* was grown in the stool. During hospitalization, the patient developed fever with a peak body temperature of 40°C, and blood cultures were positive for *Escherichia coli* (*E. coli*). Following the initiation of targeted antimicrobial therapy with levofloxacin, symptoms improved significantly and body temperature returned to normal range. Later, the microscopic examination of the plaques demonstrated that MG bodies from both the colon and the sacrum are expected in malakoplakia. These would be less commonly seen in cases isolated from both soft tissue and bone simultaneously. Malakoplakia may affect the digestive tract, and subsequently involve nearby organs such as the sacrum. This case serves as a reminder to consider malakoplakia as a differential diagnosis in the evaluation of suspected malignancies in patients of all ages. In the previously reported cases, treatment consisted of antibiotics with immunosuppressive therapy, and prednisone was adjusted. Specific antibiotic therapy with levofloxacin resulted in complete recovery without recurrence.

## Conclusion

The present case report presents the potentiality of malakoplakia induction due to malakoplakia. Numerous knowledge lacunae persist in our contemporary comprehension of the intricate underlying pathogenic pathways of malakoplakia in SLE. Physicians should invariably bear in mind that infection and granulomatous lesion can be elicited while managing symptoms in SLE patients.

## Data Availability

The original contributions presented in the study are included in the article/**Supplementary Material**. Further inquiries can be directed to the corresponding author/s.
